# Relationship Seekers Versus Relationship Selectors: Influence of Residential Mobility on How to Evaluate Others

**DOI:** 10.3389/fpsyg.2021.769487

**Published:** 2022-01-03

**Authors:** Yuchen Fang, Masato Nunoi, Asuka Komiya

**Affiliations:** ^1^Graduate School of Humanities and Social Science, Hiroshima University, Higashihiroshima, Japan; ^2^School of Human Sciences, Sugiyama Jogakuen University, Nisshin, Japan

**Keywords:** residential mobility, impression formation, socio-ecological approach, warmth, competence

## Abstract

The present study examined the effect of residential mobility on impression formation. In the study, participants were first engaged in a residential mobility priming task where they were asked to imagine and describe either frequent moving life (high-mobility condition) or less frequent moving life (low-mobility condition). They then evaluated their attitudes toward four types of target persons: competent vs. incompetent and warm vs. cold. As a result, in the high-mobility condition, the effect of competence was observed only when participants evaluated a warm person, whereas in the low-mobility condition, it appeared only when participants evaluated a cold person. The potential influence of individual residential mobility on the relationship formation is also discussed.

## Introduction

Residential mobility—that is, the frequency of moves (Oishi, 2010)—is a key socio-ecological factor leading to substantial changes in physical and social environments. In the last decade, many studies have demonstrated how residential mobility is associated with people’s psychological processes (Oishi and Talhelm, 2012; Choi and Oishi, 2020), such as self-concept (e.g., Oishi et al., 2007a), group membership and in-group cooperation (e.g., Oishi et al., 2007b, 2009), personality (e.g., Oishi and Schimmack, 2010; Jokela, 2021), and physical and mental health (e.g., Fowler et al., 2015; Hendriks et al., 2016). Although such research has revealed its influence on personal processes (i.e., How do people recognize themselves?) or social processes (i.e., What kinds of social relationships and networks do people construct? How do people behave to do so?), there has been less research exploring basic cognitive processes. How do residential moves affect people’s perceptions or attention to information? In the current study, focusing on impression formation, we explored how residential moves affect information processing.

### Two-Step Process of Impression Formation

Many studies have argued that individuals evaluate other people with two fundamental dimensions: “warmth” and “competence” (Fiske et al., 2007; Cuddy et al., 2008). Specifically, when encountering a new person, we are likely to infer their intent (e.g., “Are they good persons?”). This dimension is called *the warmth dimension* and comprises traits, such as friendliness, helpfulness, and trustworthiness. At the same time, we also aim to determine their capability to pursue and enact their intent. This dimension is referred to as *the competence dimension* and comprises traits, such as intelligence, creativity, and efficacy.

Typically, warmth judgments have priority over competence judgments when people develop others’ impressions (see Wojciszke, 2005 and Fiske et al., 2007 for review). According to Cuddy et al. (2008), the priority of warmth judgments makes sense from an evolutionary perspective because whether another person has a good or bad intent is more likely to contribute to survival than whether the other person can act on those intentions. Some studies have shown that warmth has a greater impact on the global impressions of others (especially strangers) than competence (e.g., Singh and Tor, 2008). Singh and Tor (2008) reported that the effect of likability (here, it is equivalent to warmth) on attractiveness was twice as large as competence. Other studies have shown that warmth is possibly judged before competence. Wojciszke et al. (1998), for example, reported higher accessibility of warmth than competence traits. They also found that warmth information mainly determined positivity-negativity impressions of fictitious persons, and competence information only weakly modifies them. In sum, these studies suggest the possibility that, when forming impressions of others, we go through a two-step process—first, the warmth judgment and then, the competence judgment.

However, some studies have argued that the impression-formation process depends on the perceiver’s goals and interests and that, if competence is relevant to the perceiver’s goals, competence information can strongly influence the overall impression (Wojciszke and Abele, 2008; Carrier et al., 2019). Carrier et al. (2019) conducted a series of scenario experiments and showed that competent people were more likely to be preferred in situations where cooperation was needed while the opposite was true in situations where competition occurred. Moreover, this pattern was obtained only when participants had a strong motivation to succeed. When participants were less motivated to succeed, the competence effect was not observed. These studies suggest that the influence of competence depends on people’s goals and interests. If it is irrelevant to people’s goals, its judgment can be ignored.

### Residential Mobility and Impression Formation

In this study, we explored the possibility that individuals’ residential mobility can influence the way information is processed to form an impression. Specifically, we argue that, because residential mobility determines what kind of people are preferred (or should be avoided) as a partner, it may determine what kind of people are focused on. Moreover, as discussed, it is possible that the information of the focal person is processed to the end while the information of others is not (Carrier et al., 2019). In combination, residential mobility might constrain when people proceed to the second step of the impression-formation process—that is, competence judgment.

#### Frequent Movers as “Relationship Seekers” Focus on Warm-Natured Individuals

Previous studies have argued that, although frequently moving to a new residence can bring fresh and exciting experiences, the process of relocation is also costly and can deprive individuals of local social capital (Glaeser et al., 2002; Magdol and Bessel, 2003). Therefore, a mobile lifestyle causes individuals to experience more loneliness and anxiety in their relationships (Oishi et al., 2012) and strongly motivates them to expand their social networks in new environments (Oishi et al., 2013). When trying to expand social networks, what strategies should people use? It is likely that people investigate whether a target person is good-natured or not. Once they find a good-natured person who may be a prospective partner, they take the next step and investigate the good-natured person further, including their competence, to determine whether they should develop a new relationship with them. On the other hand, if they judge that the person is not a potential partner, they may simply ignore that person from that moment, saving time, and cognitive resources.

Some studies have shown that frequent movers prefer individuals who are good to them. For example, Lun et al. (2012) found that frequent movers preferred an egalitarian helper (one who helps strangers as well as friends) to a “loyal” helper (one who helps close friends but not strangers) while less frequent movers did not show such patterns. This makes sense, because frequent movers, who are surrounded by unfamiliar people in a new environment, must rely on strangers and would like a person who helped them (strangers) more than a person who did not. Another series of studies found that people like a slightly smiling face (i.e., a face that is friendly to everyone) more when they are led to think of a mobile lifestyle than when they are led to think of a stable lifestyle (Oishi and Miao, 2013). These studies collectively indicate that frequent movers are more likely to focus on people with a warm nature (i.e., who may help them) compared to people with a cold nature when expanding their social network.

#### Less Frequent Movers as “Relationship Selectors” Focus on Cold-Natured Individuals

In contrast, less frequent movers generally have predetermined, stable relationships (Ho et al., 2006; Oishi, 2010). They do not necessarily have to enlarge their social networks. Rather, they would be more concerned with maintaining their existing social network as “safe.” To do so, they should make efforts to detect and avoid potential enemies.

Consequently, when presented with not-well-known individuals, they are more likely to focus on those with cold natures who may be potential harm-doers. Once they notice that a person is dangerous at the first step of impression formation, they will try to learn more about the person to avoid him or her effectively. Through this process, less frequent movers might take other information, such as competence into account—that is, they might proceed to the second step of the two-step process of impression formation. On the other hand, if the person is not dangerous, the second step might be skipped. The most important thing is that a person is not an enemy.

The most relevant studies were conducted by Glenn Adams, who presented the concept of “cautious intimacy” (e.g., Adams and Plaut, 2003; Li et al., 2015). Originally, Ghanaians were found to be more likely than Americans to see enemies in their social ties and hesitate to enlarge social networks. The researchers argued that Ghanaians are embedded within their tight-knitted social networks and cannot freely choose friends as they like (Adams and Plaut, 2003; Adams, 2005). This point was recently supported by Li et al. (2015), who showed that individuals with lower relational mobility (i.e., people who have less opportunity to establish new relationships) reported that they are more cautious and vigilant toward friendships than individuals with high relational mobility (i.e., people who have more opportunity to establish new relationships). This should be the case for a newly met person. Whether the person already exists in their social network or not, low-mobility people will focus on and be careful concerning a potential enemy: a cold person.

### The Current Study

This study examined whether residential mobility influences the process of impression formation. To achieve this, we used a mobility priming task (Oishi et al., 2012, 2013). In the task, participants were asked to imagine either a frequent moving life or less frequent moving life. It is known that the thought of a mobile lifestyle successfully evokes anxiety associated with moving and is effective in manipulating residential mobility. We believe that it is the best way to directly examine how residential mobility influences impression formation.

As dependent variables, we assessed attitude toward the target person as well as the overall impression (i.e., likability). We explored attitude because it is also key to determining interpersonal behavior. A positive attitude motivates people to approach a target while a negative attitude motivates people to avoid it. In this sense, attitudes and impressions function similarly, and attitude seems to be formed based on the same information as the impression. Therefore, following the tripartite model of attitude (Rosenberg and Hovland, 1960; Breckler, 1984), we also measured emotional, behavioral, and cognitive aspects of attitude toward a person as well as overall impression.

Assuming (i) that competence judgment is executed only when the target person matters, and (ii) who matters differ between individuals with high- and low-residential mobility, we hypothesized that the situation in which the competence judgment is made depends on residential mobility—that is, we expected a three-way interaction effect across residential mobility, warmth, and competence dimensions. Specifically, people in the high-mobility condition are more likely to form positive impressions and attitudes with a person who is competent than incompetent, only when the target is a potential partner (i.e., the target is a warm person). When the target does not seem to be a potential partner and is not worth forming a relationship with (i.e., the target is a cold person), they are not involved in the competence judgment. On the other hand, among people in the low-mobility condition, such competence effect is found only when the target is a cold person. This is because people with the low-residential mobility would be cautious with, focus on, and analyze the potential enemy. When the target is good-natured and will not pose any threat (i.e., the target is a warm person), again, they are not engaged in the competence judgment for saving cognitive resources.

To date, no studies have examined the influence of residential mobility on impression formation. With the increasing rate of migration globally today, it is essential to explore the impact of residential moves on psychological tendencies (Choi and Oishi, 2020). In particular, the process of impression formation has great significance in relationship formation in our social lives. Depending on the impression formed, individuals can choose to strengthen or avoid relationships with others. It is hoped that this research will contribute to a deeper understanding of how ecological factors influence a basic cognitive process, and more generally, people’s psychology and behaviors.

## Materials and Methods

### Participants and Design

Seventy-four Japanese students taking psychology courses participated in this study. They received a bonus course credit as compensation for participation. Eleven participants who reported engaging in the priming task for less than 1 min or more than 10 min were considered outliers and were excluded from the following analyses. Since Google Forms cannot record or manipulate the flow of time, we asked participants to report their start and end of the priming task. The distribution was examined, and the criteria were determined (see Supplementary Material 1 for further details). Of the 63 participants (24 men and 39 women; *M*_age_ = 19.6, *SD* = 1.5), 30 were assigned to the high-mobility condition, and 33 were assigned to the low-mobility condition. The study used a 2 × 2 × 2 design with manipulation of residential mobility (high vs. low) as a between-participants factor and competence (competent vs. incompetent) and warmth dimensions (warm vs. cold) as within-participants factors.

### Stimuli

In this study, we used eight-person descriptions (i.e., two people × warm or cold × competent or incompetent; see Supplementary Material 2 for all descriptions). To select stimuli, a pilot study was conducted.

Twelve Japanese students (six men and six women; *M*_age_ = 21.3, *SD* = 0.9) participated in the pilot study. There were two parts. First, the participants were presented with 24 sentences that described a person’s behavior. Half of the behaviors represent a person’s warmth (e.g., “*When X walks on the road, X always walks on the side closer to the roadway, and takes care so that his/her friend is not hit by the car*”), whereas the other half represents a person’s coldness (e.g., “*When X was found to be lost, X left without saying thank you*”). Each sentence was presented with five adjectives related to warmth (i.e., *warm, friendly, considerate, pleasant*, and *kind*), and participants rated the extent to which each adjective could be applied to a person on a 7-point scale (1 = *not applicable at all* to 7 = *applicable very much*). Similarly, in the next part, they rated another set of 24 behavioral sentences in terms of competence, using five adjectives (i.e., *smart, intelligent, sensible, a fast learner*, and *clever*) on a 7-point scale; half of the behaviors represent a person’s competence in the university (e.g., “*X has outstanding performance in the university; all the grades are A+, except for one A*”), whereas the other half represents a person’s incompetence (e.g., “*X were not able to graduate from the university even in five years because of a lack of credits*”). In each section, all participants view sentences in the same order.

Because both warmth and competence scales showed excellent internal consistency (warmth: *α* = 0.93; competence: *α* = 0.97), the average scores were used for the selection. We selected eight warmth and eight coldness sentences, which scored the highest (*M* = 6.42–6.12) and the lowest (*M* = 1.37–1.77) in terms of the warmth scale, respectively, and eight competence and eight incompetence sentences, which scored the highest (*M* = 6.25–5.67) and the lowest (*M* = 1.63–2.33) in terms of the competence scale, respectively. The mean scores of the 32 sentences were significantly different from the midpoint (4) of the scale, *t*s > 5.17, *p*s < 0.001.

We used these 32 sentences to create a total of eight-person descriptions with two types of descriptions for each of the four conditions (i.e., warm-competent, warm-incompetent, cold-competent, and cold-incompetent). Each person description was composed of two sentences to represent a person’s warmth and two to represent a person’s competence. For example, in the warm-competent condition, we used two warmth sentences and two competence sentences to describe Target person A and two warmth sentences and two competence sentences to describe Target person B. We presented these eight-person descriptions in the person-evaluation task in our experiment.

### Procedure

Participants were engaged in an experiment on the web (Google Forms). After answering demographic questions, including sex and age, participants were engaged in the priming task for 5 min (Oishi et al., 2012). They were randomly assigned to one of two residential mobility conditions (high vs. low). In both conditions, participants were asked to imagine a situation where they got a job that they had always wanted and described what their life would be like in the situation as much as possible. In the high-mobility condition, participants read that the job required them to move to a new place every other year, whereas those in the low-mobility condition read that the job required them to stay in the same place for 10 years. For the manipulation check, they then rated two questions (Li et al., 2019): “How often do you think you will move in the life you have just imagined?” and “How often do you think you will move in the future in the life you have just imagined?” using a 6-point scale (1 = *very infrequently* to 6 = *very frequently*).

**Figure 1:**
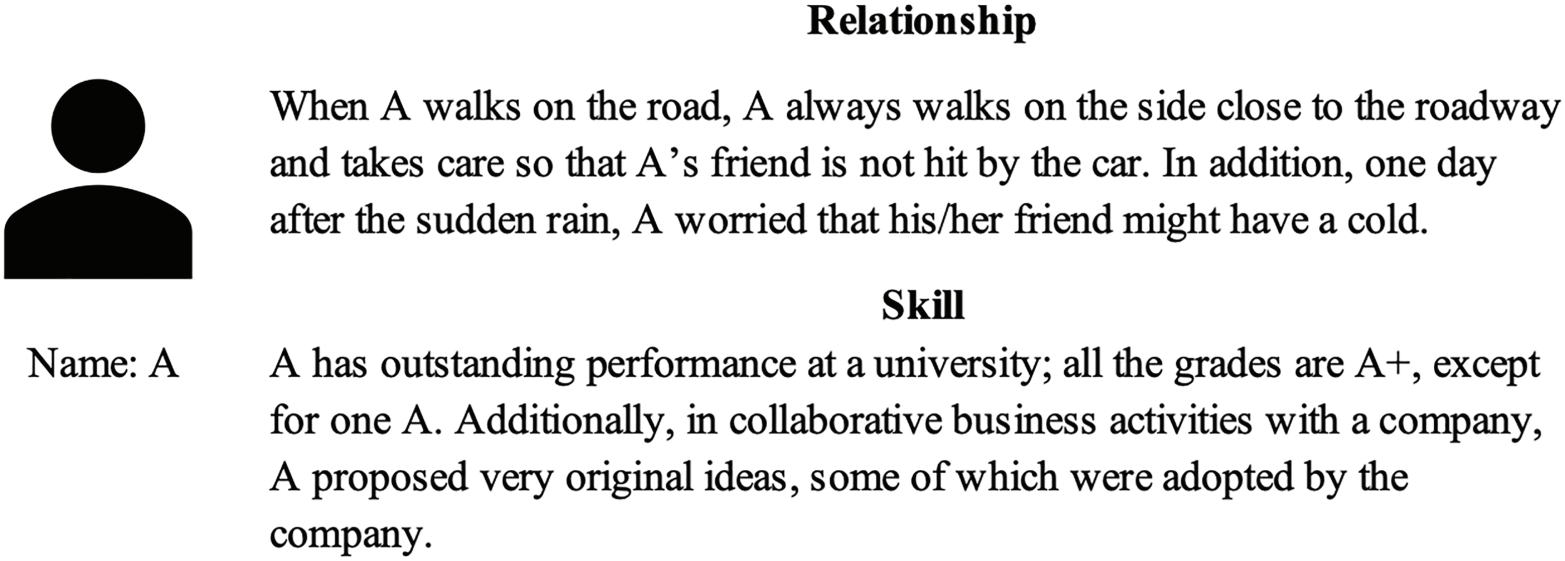
An example of stimuli.

Participants were then engaged in a person-evaluation task. They read a person description (see Figure 1 for an example) and rated their impressions and attitudes toward the person using a 6-point scale (1 = *not at all* to 6 = *extremely*). Specifically, they first rated the extent to which they would like the target in general (*likability*). After the likability rating, they rated to what extent they would do each of interpersonal behaviors with the target with six items (*behaviors*; “I want to get along with him/her,” “I want to have a talk with him/her when I have worries,” “If she/he is in trouble, I am willing to offer help,” “I want to ask an important work,” “I want to do the class assignment together,” and “I want to hear advice on my class assignment”). They then rated to what extent they would feel each of emotions toward the target with 12 items (*emotions*), which were classified into four subscales (Ogihara et al., 2013): “happy” and “satisfied” (*general positive emotions; GPE*); “friendly,” “proud,” “superior,” and “respect” (*interpersonal positive emotions; IPE*); “boredom,” “angry,” “frustration,” and “depression” (*general negative emotions; GNE*); and “nervous” and “hostile” (*interpersonal negative emotions; INE*). In the original work, INE includes “lonely,” “sad,” “guilt,” and “self-blame” in addition to the two items used in the current study. We did not examine these four emotions because we usually did not feel them in a stranger. Finally, they were asked to rate the extent to which they felt that their relationship with the target was important (*cognition; relationship importance; and cognitive-based attitude*). There were eight-person descriptions (i.e., two people × warm or cold × competent or incompetent), and they were presented in a fixed order.

After the experiment, participants were thanked and debriefed. This experimental procedure received the approval of the Ethics Committee of Hiroshima University for the protection of human participants (approval number 02-32) and all participants gave their written informed consent prior to starting the experiment.

## Result

### Manipulation Check

We performed *t*-tests on the manipulation check items to assess whether mobility was successfully manipulated. The results showed that participants in the high-mobility condition were more likely to perceive frequent moves both in the life imagined than those in the low-mobility condition, *M*_high_ = 3.67 (*SD*_high_ = 1.84) vs. *M*_low_ = 2.12 (*SD*_low_ = 1.11), *t*(46.69) = 3.98, *p* < 0.001, *d* = 1.01, and in the future life, *M*_high_ = 3.63 (*SD*_high_ = 1.67) vs. *M*_low_ = 2.52 (*SD*_low_ = 1.18), *t*(51.52) = 3.04, *p* = 0.004, *d* = 0.77. These results confirmed that the manipulation was successful.

### Likability (Overall Impression)

**Figure 2:**
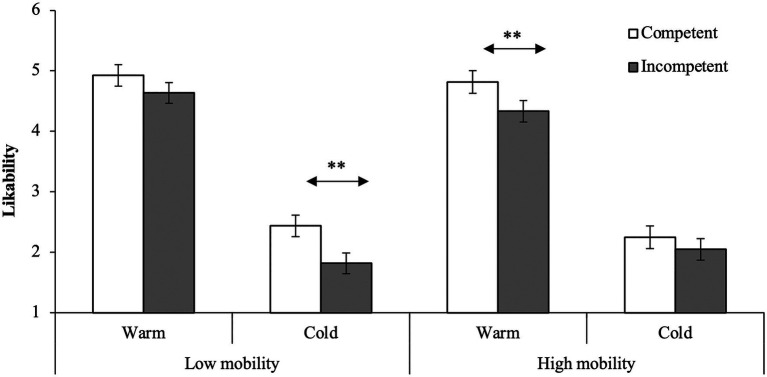
Means and SEs on likability. **p < 0.01.

To test the hypothesis, we first conducted a 2 (residential mobility: high vs. low) × 2 (competence: competent vs. incompetent) × 2 (warmth: warm vs. cold) mixed-design ANOVA on likability. The pertinent means and standard errors are shown in Figure 2. First, not surprisingly, the main effects of competence and warmth were significant, *F*(1, 61) = 14.13, *p* < 0.001, partial *η*^2^ = 0.19 and *F*(1, 61) = 415.98, *p* < 0.001, partial *η*^2^ = 0.87, respectively. Not surprisingly, participants were more likely to prefer competent people than incompetent people and warm people than cold people. The main effect of residential mobility was not significant, *F*(1, 61) = 0.42, *p* = 0.521, partial *η*^2^ = 0.01. In addition, there were no significant two-way interaction effects, *F*s < 0.83, *p*s > 0.366.

Most importantly, as expected, we observed a significant three-way interaction effect, *F*(1, 61) = 4.85, *p* = 0.031, partial *η*^2^ = 0.07. *Post-hoc* analysis (Holm method) showed that participants in the high-mobility condition were more likely to prefer the competent than the incompetent when the target was a *warm* person, *F*(1, 122) = 6.92, *p* = 0.010. This pattern was not observed when the target was a cold person, *F*(1, 122) = 1.18, *p* = 0.279. On the other hand, in the low-mobility condition, participants were more likely to prefer the competent than the incompetent when the target was a *cold* person, *F*(1, 122) = 12.57, *p* = 0.001. Again, this tendency was not shown if the target was a warm person, *F*(1, 122) = 2.70, *p* = 0.103. Overall, the results showed a pattern consistent with our predictions.

### Emotions

We conducted the same mixed-design ANOVAs on GPE, IPE, GNE, and INE. The internal consistency for each scale was acceptable (GPE: *r* = 0.71–0.92, IPE: *α* = 0.59–0.71, GNE: *α* = 0.71–0.83), except for INE (*r* = 0.12–0.51). Because Ogihara et al.’s (2013) sample size was larger than ours (*n* = 93), its interpretation should be more valid than ours. Therefore, following their procedure, we averaged the scores by category and submitted them to the analysis. The pertinent means and standard deviations are listed in Table 1.

**Table 1:** Means and standard deviations of ratings in each condition.

Measure	Mobility condition	Warm				*p*	Cold				*p*
		Competent		Incompetent			Competent		Incompetent		
**Emotion-based attitude**											
General positive	High	4.23	(1.08)	3.50	(0.83)	<0.001	^***^1.81	(0.80)	1.70	(0.86)	0.465
	Low	3.89	(1.12)	3.86	(0.90)	0.788	1.86	(0.87)	1.64	(0.63)	0.123
Interpersonal positive	High	4.00	(0.57)	3.41	(0.66)	<0.001	^***^2.16	(0.75)	1.96	(0.71)	0.150
	Low	3.84	(0.93)	3.67	(0.66)	0.218	2.19	(0.80)	1.75	(0.57)	<0.001^***^
General negative	High	1.68	(0.70)	2.06	(0.67)		2.75	(0.97)	2.90	(1.01)	
	Low	1.87	(0.67)	1.88	(0.53)		3.00	(0.95)	3.16	(0.93)	
Interpersonal negative	High	2.68	(0.85)	1.85	(0.68)		2.63	(0.92)	2.23	(0.77)	
	Low	2.64	(0.85)	1.78	(0.59)		3.03	(1.25)	2.55	(1.21)	
Behavior-based attitude	High	5.07	(0.80)	3.65	(0.69)		2.38	(0.89)	1.81	(0.70)	
	Low	4.77	(1.01)	3.77	(0.71)		2.59	(1.12)	1.89	(0.63)	
Relationship importance	High	5.18	(0.89)	4.37	(0.93)	<0.001	^***^2.65	(1.23)	2.52	(1.34)	0.466
	Low	4.86	(1.05)	4.65	(1.01)	0.225	2.55	(1.39)	2.29	(1.17)	0.142

*** *p < 0.001*.

#### Positive Emotions

According to the GPE and IPE, almost the same pattern was observed. First, the main effects of competence, *F*(1, 61) = 14.38, *p* < 0.001, partial *η*^2^ = 0.19 for GPE and *F*(1, 61) = 27.02, *p* < 0.001, partial *η*^2^ = 0.31 for IPE, and warmth, *F*(1, 61) = 233.28, *p* < 0.001, partial *η*^2^ = 0.79 for GPE and *F*(1, 61) = 310.64, *p* < 0.001, partial *η*^2^ = 0.84 for IPE, were significant. Unsurprisingly, participants were more likely to experience GPE and IPE toward competent people than incompetent and warm people than cold people. Neither the main effect of residential mobility nor the two-way interaction effects were significant, *F*s < 2.56, *p*s > 0.115, except for the residential mobility × competence interaction effect on GPE, *F*(1, 61) = 4.00, *p* = 0.050, partial *η*^2^ = 0.06.

Most importantly, we observed a significant three-way interaction effect, *F*(1, 61) = 8.62, *p* = 0.005, partial *η*^2^ = 0.12 for GPE and *F*(1, 61) = 8.03, *p* = 0.006, partial *η*^2^ = 0.12 for IPE. In the high-mobility condition, we obtained the same pattern as likability. That is, participants were more likely to experience GPE and IPE toward the competent than the incompetent when the target was a *warm* person, *F*(1, 122) = 25.61, *p* < 0.001 for GPE and *F*(1, 122) = 20.97, *p* < 0.001 for IPE, but not when the target was a cold person, *F*(1, 122) = 0.57, *p* = 0.451 for GPE and *F*(1, 122) = 2.40, *p* = 0.124 for IPE. On the other hand, in the low-mobility condition, there were somewhat different patterns. For IPE, as expected, the competence effect was obtained when the target was a *cold* person, *F*(1, 122) = 12.72, *p* = 0.001, but not when the target was a warm person, *F*(1, 122) = 1.75, *p* = 0.189. However, for GPE, the competence effect was not significant either when the target was a warm or cold person, *F*(1, 122) = 0.08, *p* = 0.782 and *F*(1, 122) = 2.59, *p* = 0.110, respectively.

#### Negative Emotions

According to GNE and INE, not surprisingly, the main effects of competence, *F*(1, 61) = 6.20, *p* = 0.016, partial *η*^2^ = 0.09 for GNE and *F*(1, 61) = 76.98, *p* < 0.001, partial *η*^2^ = 0.56 for INE, and warmth, *F*(1, 61) = 100.75, *p* < 0.001, partial *η*^2^ = 0.62 for GNE and *F*(1, 61) = 11.31, *p* = 0.001, partial *η*^2^ = 0.16 for INE, were significant. Participants were more likely to experience GNE and INE toward cold people than warm people. As for competence, however, the directions were different between the GNE and INE. Participants were more likely to experience GNE toward incompetent people than toward competent people, while they were more likely to experience INE toward competent people than incompetent people. Considering the difference between INE and GNE, the different directions might not be surprising. Interpersonal emotions represent interpersonal distance (“whether do I approach or avoid the person?”), whereas general emotions are likely to represent a comprehensive evaluation. Because competent-cold people are especially dangerous and should be disengaged, INE might be assessed higher for them than for incompetent-cold people. On the other hand, because participants were more likely to find negative traits in incompetent-cold people than competent-cold people, GNE might be assessed higher for them.

No other effects were significant, *F*s < 3.38, *p*s > 0.071, except for the competence × warmth interaction effect on INE, *F*(1, 61) = 7.46, *p* = 0.008, partial *η*^2^ = 0.34. That is, participants reported stronger INE for the competent than the incompetent when the target was either warm or cold person, *F*(1, 122) = 65.86, *p* < 0.001 and *F*(1, 122) = 17.94, *p* < 0.001, but the effect was greater for warm people than cold people. In sum, for negative emotions, unlike positive emotions, we could not find the expected pattern.

### Behaviors

Next, we conducted the same mixed-design ANOVA on behavior-based attitude. Since internal consistency was acceptable (*α* = 0.75–0.93), the averaged score was submitted to the analysis. The pertinent means and standard deviations were shown in Table 1. First, the main effects of competence and warmth were significant, *F*(1, 61) = 89.74, *p* < 0.001, partial *η*^2^ = 0.60 and *F*(1, 61) = 386.90, *p* < 0.001, partial *η*^2^ = 0.86, respectively, and the main effect of residential mobility was not significant, *F*(1, 61) = 0.04, *p* = 0.845, partial *η*^2^ = 0.001. Participants were more willing to interact with competent people than incompetent people and warm people than cold people. In addition, there were no significant interaction effects, *F*s < 3.60, *p*s > 0.063, except for the warmth × competence interaction effect, *F*(1, 61) = 15.90, *p* < 0.001, partial *η*^2^ = 0.21. That is, participants were more willing to interact with the competent than the incompetent among both warm and cold people, *F*(1, 122) = 99.71, *p* < 0.001 and *F*(1, 122) = 27.45, *p* < 0.001, but the effect was greater for warm people than cold people. This pattern is similar to INE.

### Cognition (Relationship Importance)

Finally, we conducted the same mixed-design ANOVA on relationship importance. The pertinent means and standard deviations are listed in Table 1. As with other dependent variables, the main effects of competence and warmth were significant, *F*(1, 61) = 16.01, *p* < 0.001, partial *η*^2^ = 0.21 and *F*(1, 61) = 218.06, *p* < 0.001, partial *η*^2^ = 0.78, respectively; the main effect of residential mobility was not significant, *F*(1, 61) = 0.19, *p* = 0.665, partial *η*^2^ = 0.003. Participants were more likely to think that the relationship was important when the partner was competent than incompetent and when the target was warm than cold. In addition, there were no significant two-way interaction effects, *F*s < 3.72, *p*s > 0.059.

Here, the expected three-way interaction effect was significant, *F*(1, 61) = 4.85, *p* = 0.031, partial *η*^2^ = 0.07. Participants in the high-mobility condition were more likely to think that the relationship is important when the partner was a competent*-warm* person than when the partner was an incompetent-*warm* person, *F*(1, 122) = 21.64, *p* < 0.001. This pattern was not observed if the target was a cold person, *F*(1, 122) = 0.58, *p* = 0.449. On the other hand, in the low-mobility condition, the competence effect was not significant when the target was a warm or cold person, *F*(1, 122) = 1.61, *p* = 0.207 and *F*(1, 122) = 2.37, *p* = 0.126, respectively. This pattern is similar to that of GPE.

### *Post-hoc* Statistical Power Analysis

Our primary finding was that, in the high-mobility condition, the competence effect was obtained only when the target was a warm person while, in the low-mobility condition, the competence effect was obtained only when the target was a cold person (i.e., the mobility × competence × warmth interaction effect). As reported in each section, this three-way interaction effect was associated with medium to large effect size (partial *η*^2^ = 0.07 in likability and relationship importance and partial *η*^2^ = 0.12 in GPE and IPE). To evaluate the reliability of the findings, we used G^*^Power 3.1.9.6 (Erdfelder et al., 1996) to obtain *post-hoc* power based on effect sizes (*f* = 0.27 in likability and relationship importance, *f* = 0.37 in GPE and IPE), sample sizes (*n* = 63), and alpha level (alpha = 0.05). The estimated power exceeded 0.99. Thus, the primary finding of this study was associated with a satisfactory level of reliability.

## Discussion

We aimed to examine how residential mobility influences impression formation. To achieve this, we used a priming task (Oishi et al., 2013) and investigated the effect of residential mobility on impressions as well as attitudes. The results partially support our hypothesis. In the high-mobility condition, participants were more likely to prefer, feel positive with, and attach importance to relationships with the competent than the incompetent, particularly when the target was a *warm* person. In contrast, in the low-mobility condition, this competence effect was shown, particularly when the target was a *cold* person, although some measures did not show this pattern. Overall, these findings provide evidence of the influence of residential mobility on the impression-formation process. We discuss each type of measurement below.

### Overall Impression

In the current study, people who imagined a life of frequent moving were more likely to respond positively to competent people than to incompetent people when they were warm. In contrast, when the target was a cold person, there was no competence effect. On the other hand, people who imagined a life of less frequent moving showed a competence effect only when the target was a cold person. These findings are consistent with our hypothesis, suggesting that people’s focus depends on residential mobility. That is, people with high residential mobility, who are strongly concerned about forming new relationships with desirable partners (Oishi et al., 2012, 2013), are more likely to focus on a warm person while people with low-residential mobility, who would like to avoid contact with the enemy, are more likely to focus on a cold person. In this sense, this is the first study to demonstrate that residential mobility can influence the evaluation of others.

### Emotions

As for positive emotions, the pattern was almost consistent with the overall impression. Participants in the high-mobility condition were more likely to feel positive emotions toward competent people than incompetent people only when they were warm. On the other hand, participants in the low-mobility condition were more likely to feel positive emotions (especially interpersonal positive emotions) toward competent people than incompetent people only when they were cold. Again, this pattern was consistent with our hypothesis, suggesting the possibility that residential mobility constrains who is focused on and whose information is processed. Note that, in the low-mobility condition, the competence effect on general positive emotions, such as happiness and satisfaction, did not reach statistical significance. We discuss this further in the cognition section.

However, we did not find any influence of residential mobility on negative emotions. This may be because we usually feel such negative emotions in the process of relationship *maintenance*, not *formation*. That is, because such negative emotions typically result from one’s failure to have smooth, “ongoing” relationships (e.g., I caused trouble for him; Kitayama et al., 2000), they may be relatively unrelated to the current study, which is concerned with developing new relationships. It will be promising to compare how negative emotions work in relationship maintenance vs. formation situations.

### Behaviors

Additionally, we found no influence of residential mobility on behavior. This may have resulted from the fact that we used items that require competence, such as working together and seeking help. Because of this, participants might simply judge whether the target person would help them in the short term, not whether they would like to form a relationship with the target in the long term. Further research is needed with a variety of behavioral items (e.g., being a roommate), which suggests a deep, long-term relationship.

### Cognition

Regarding cognition-based attitude, relationship importance, the pattern was the same as for general positive emotion. Participants in the high-mobility condition were more likely to evaluate the relationship as important with competent people than incompetent people only when they were warm. On the other hand, there were no significant effects in the low-mobility condition whether the target was warm or cold. This pattern may have emerged because the competence effect in the low-mobility condition may reflect a tendency to try to find a good point for the target in some way. That is, because they cannot break the relationship (due to the low-residential mobility), people may only justify the value of their partners. Therefore, the competence effect was observed in the value of the target (i.e., likability and IPE), but not in the value of the relationship in general (i.e., GPE and relationship importance). Future studies should explore this possibility.

### Limitation

Although the study reveals the effect of residential mobility on relationship formation, it has several limitations. First, although we used a priming task to manipulate residential mobility, this might have caused the problem of ecological validity. It is important to conduct a survey to investigate whether frequent movers evaluate competence only when they find a potential friend while non-movers will do so when finding a potential enemy. Second, we found some variations in the competence effect, particularly in the low-mobility condition. Future studies should address why we found competence-warmth interactions in some items but not in others. Despite these limitations, we found a pattern that was somewhat consistent with our hypothesis.

## Conclusion

The current study examined a novel prediction that residential moves would influence how individuals evaluate others, which is the basic process of forming relationships. These findings have many direct implications for interpersonal strategies, personal preferences, cognitive tendencies, and more. Further studies on residential mobility will help understand how socio-ecological factors explain and predict human behaviors and psychological processes.

## Data Availability Statement

The data associated with this study are available on the Open Science Framework: https://osf.io/2pj9e/.

## Ethics Statement

This experimental procedure received the approval of the Ethics Committee of Hiroshima University for the protection of human participants (approval number 02–32) and all participants gave their written informed consent prior to starting the experiment.

## Author Contributions

YF and MN contributed to conception and design of the study and performed the statistical analysis. YF and AK wrote the manuscript. All authors contributed to manuscript revision, read, and approved the submitted version.

## Funding

This research was supported by JSPS KAKENHI grant number 18 K13270 to AK. The funders had no role in study design, data collection and analysis, decision to publish, or preparation of the manuscript.

## Conflict of Interest

The authors declare that the research was conducted in the absence of any commercial or financial relationships that could be construed as a potential conflict of interest.

## Publisher’s Note

All claims expressed in this article are solely those of the authors and do not necessarily represent those of their affiliated organizations, or those of the publisher, the editors and the reviewers. Any product that may be evaluated in this article, or claim that may be made by its manufacturer, is not guaranteed or endorsed by the publisher.

## Supplementary Material

The Supplementary Material for this article can be found online at: https://www.frontiersin.org/articles/10.3389/fpsyg. 2021.769487/full#supplementary-material

## Abbreviations

GPE: General Positive Emotion
IPE: Interpersonal Positive Emotions
GNE: General Negative Emotions
INE: Interpersonal Negative Emotions
