# Supplementary material for: Relationship Seekers Versus Relationship Selectors: Influence of Residential Mobility on How to Evaluate Others
Source: Front Psychol. 2022 Jan 3;12:769487. doi: 10.3389/fpsyg.2021.769487 (PMC8762166; doi:10.3389/fpsyg.2021.769487)
Supplement: Supplementary file 1 [file Data_Sheet_1.DOCX]

Supplementary Material

# Distribution of self-reported time engaging in the priming task.

We instructed participants to engage in the priming task for 5 min. Since Google Forms cannot record or manipulate the flow of time, we asked participants to report their start and end of the priming task. We calculated the engaging time by subtracting the start time from the end time. The distribution is shown in Supplementary Figure 1.


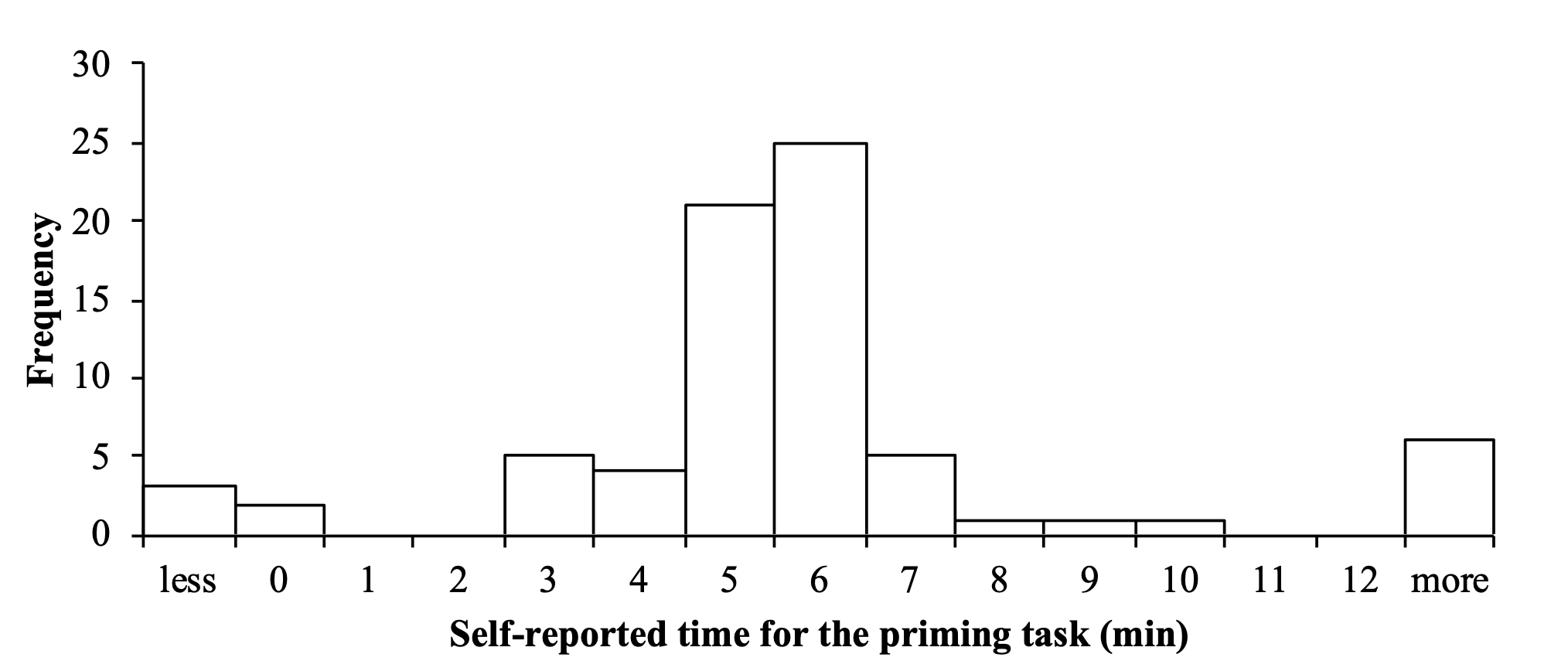


**Supplementary Figure 1.** Frequency of self-reported time for the priming task.

We determined the exclusion criteria in two ways. First, we excluded five participants who reported engaging in the priming task for less than 1 min (i.e. two people who reported zero minutes and three people who reported “minus” minutes), since they were considered as not following the instruction appropriately. Second, for the same reason, we excluded six participants who reported more than 10 min (i.e. participants who reported engaging in the task for 28 to 1,093 min). Accordingly, we analysed the data provided by the participants who reported engaging in the priming task for 3 to 10 min (n = 63; 85.1%).

# All stimuli of the person evaluation task.

For each target person, information about the relationships and skills was presented. There were eight-person descriptions (i.e. two people × warm or cold × competent or incompetent). They were presented in a fixed order. All stimuli are shown below.

## Warm and competent target

Person A

Relationship: When A walks on the road, A always walks on the side close to the roadway and takes care so that A’s friend is not hit by the car. In addition, one day after the sudden rain, A worried that his/her friend might have a cold.

Skill: A has outstanding performance at a university; all the grades are A+, except for one A. Additionally, in collaborative business activities with a company, A proposed very original ideas, some of which were adopted by the company.

Person B

Relationship: B is very popular in class because B has a friendly personality and likes playing with friends. In addition, B gave half of his/her lunch to his/her classmates who did not know about the temporary closure of the cafeteria.

Skill: B has high self-learning ability. When B was in the first year in the university, B acquired a qualification that students could normally get in the third year. In addition, B’s research ability was high. His/her graduation thesis was selected as the best graduation thesis.

## Warm and incompetent target

Person C

Relationship: When C’s friend spilled a drink on his/her white shirt, C lent his/her jacket to his/her friend. Earlier, C bought his/her friend’s favourite cake to cheer him/her up after he/she lost his/her pet.

Skill: C was not able to graduate from the university even in five years because of a lack of credits. In addition, C was not able to propose any good ideas for the alumni association event because C did not prepare at all in advance.

Person D

Relationship: When friends ask D for advice, D always listens empathically to their problems and gives useful advice. In addition, when walking along the street, D always lets everyone know if a car comes from behind.

Skill: Although D is in the third year in the university, D is not able to understand junior-high-school-level English. In addition, D is always late for class, even if the professor warns him/her.

## Cold and competent target

Person E

Relationship: The other day, when E was found to be lost, E left without saying thank you. In addition, whenever E works with a team in the university, E only performs the work given to him/her and does not help other people on the same team.

Skill: E has received a commendation from the school because of his/her excellent performance every year. In addition, E has high practical skills and contributes significantly to the success of many activities at the alumni association.

Person F

Relationship: F is a person who does not give a hand even if his/her friend falls next to him/her. F also does not greet classmates. F only provides a curt answer, even if spoken to.

Skill: F won first prize in the English speech contest in school. In addition, F was tentatively appointed as a famous global company.

## Cold and incompetent target

Person G

Relationship: G does not cooperate with his/her group members on assignments that are not of his/her interest. In addition, G’s attitude towards his/her friends who have trouble is unkind.

Skill: G has not passed a compulsory course, although he/she has taken it thrice. In addition, G’s school grades were so bad that he/she could not answer the question teachers asked in the class properly.

Person H

Relationship: A few days ago, a college freshman asked H for directions. However, H thought it was annoying, so H said that he/she did not know. Even if H sees a person in trouble, H pretends not to know and passed by without talking to them.

Skill: H always makes mistakes in his/her part-time jobs, and H has been fired three times. H has lent an apartment near the university for a long time. However, H often forgets to pay electricity bills, resulting in a power cut.
